# LncRNA RP11-499E18.1 Inhibits Proliferation, Migration, and Epithelial–Mesenchymal Transition Process of Ovarian Cancer Cells by Dissociating PAK2–SOX2 Interaction

**DOI:** 10.3389/fcell.2021.697831

**Published:** 2021-09-21

**Authors:** Juan Yang, Shuping Peng, Keqiang Zhang

**Affiliations:** ^1^Department of Gynecologic Oncology Ward 5, Hunan Cancer Hospital, The Affiliated Cancer Hospital of Xiangya School of Medicine, Central South University, Changsha, China; ^2^NHC Key Laboratory of Carcinogenesis of Hunan Cancer Hospital, The Affiliated Cancer Hospital of Xiangya School of Medicine, Central South University, Changsha, China; ^3^The Key Laboratory of Carcinogenesis and Cancer Invasion of the Chinese Ministry of Education, Cancer Research Institute, Central South University, Changsha, China

**Keywords:** ovarian cancer, RP11-499E18.1, PAK2, SOX2, EMT

## Abstract

**Background:** Ovarian cancer (OC)is a deadly gynecological malignancy worldwide. It is urgent to identify diagnostic biomarkers of OC to disclose the underlying mechanism.

**Methods and Materials:** Bioinformatics analysis was used to identify target genes. Gene expression was detected and altered by qRT-PCR and cell transfection, respectively. The interaction between RP11-499E18.1 and PAK2, as well as that between PAK2 and SOX2, was determined using RNA pulldown, RNA immunoprecipitation (RIP), and co-immunoprecipitation (co-IP) assay, respectively. Localizations of RP11-499E18.1, PAK2, and SOX2 were respectively determined employing immunohistochemical (IHC), IF, and FISH. The regulatory effects of RP11-499E18.1, PAK2, and SOX2 on OC cell proliferation, migration, colony formation, epithelial–mesenchymal transition (EMT)-related factor expression, and SOX2 nuclear translocation were determined. Finally, the effects of RP11-499E18.1 and PAK2 expression on the tumor growth in nude mice were determined.

**Results:** RP11-499E18.1, PAK2, and SOX2 were selected in our study. RP11-499E18.1 was downregulated, while PAK2 and SOX2 was upregulated in OC tissues and cells. RP11-499E18.1 coexists in the nucleus and cytoplasm of OC cells. There is an interaction between RP11-499E18.1 and PAK2, as well as PAK2 and SOX2 in OC cells. Alteration of RP11-499E18.1 and PAK2 expression both had no influence on PAK2 and SOX2 levels, but PAK2 upregulation notably augmented p-SOX2 level. RP11-499E18.1 overexpression suppressed OC cell proliferation, migration, and colony formation, as well as SOX2 nuclear translocation. Besides, it inhibited tumor growth in nude mice. However, these effects were notably reversed by PAK2 upregulation and eventually offset by SOX2 knockdown. Additionally, RP11-499E18.1 overexpression reduced PAK2–SOX2 interaction and SOX phosphorylation, and increased the binding of RP11-499E18.1 by PAK2.

**Conclusion:** These lines of evidence demonstrated that RP11-499E18.1 might play its tumor suppressor roles in OC *via* regulation of the RP11-499E18.1–PAK2–SOX2 axis. This research indicated that RP11-499E18.1 might be used as a diagnostic biomarker for OC in the future.

## Introduction

Ovarian cancer (OC) is one of the most common and deadly gynecological malignancies in the world, which is characterized by high incidence and poor prognosis (Allemani et al., 2018; Kossaï et al., 2018). The overall 5-year survival rate of OC patients is merely 30–50%, because approximately 80% of the patients were in advanced stages at the time of diagnosis (Sundar et al., 2015; Allemani et al., 2018). The major reasons for such poor survival rate mainly lie in the inconspicuous early symptoms and limited screening approaches (Au et al., 2015; Menon et al., 2018). Considering that the outcomes of OC patients diagnosed at the early stage of OC is more satisfactory (Cress et al., 2015), identifying new clinical diagnostic biomarkers of OC with high sensitivity and accuracy and thereby obtaining a further understanding of the potential molecular regulating mechanism will be of great significance for future OC treatment.

Long non-coding RNAs (lncRNAs) are a set of ncRNAs composed of more than 200 nucleotides transcribed by RNA polymerase II (Nagano and Fraser, 2011; Huarte, 2015). In recent years, accumulating investigations have recorded that it functions as a key regulator in tumor development and progression (Hosono et al., 2017; Bill et al., 2019; Wang et al., 2020). It was disclosed that lncRNAs can exert its regulating roles in various biological functions of tumors, such as proliferation, apoptosis, and metastasis, by post-transcriptional regulation, ceRNA regulation mechanism, genomic stability, and epigenetics (Gutschner and Diederichs, 2012; Arnes et al., 2019; Tang et al., 2019). Besides, an earlier literature demonstrated that lncRNAs participated in the regulation of cellular processes depending on their location: cytoplasmic lncRNAs can affect cellular signaling cascades and regulate mRNA translation or stability, while nuclear lnRNAs are capable of transcriptional regulation, RNA processing, and chromatin interactions (Schmitt and Chang, 2016). In the last few years, the functions of many lncRNAs in OC have been investigated. For instance, it was reported that lncRNA NORAD knockdown markedly repressed the proliferation, epithelial–mesenchymal transition (EMT), migration, and invasion of OC cells (Xu et al., 2020). Similar results were also recorded in another investigation that demonstrated the tumor-suppressing roles of LINC-PINT in OC (Hao et al., 2020). Therefore, investigation of the efficacies of lncRNAs in OC may be of great significance for future treatments.

As an emerging biomedical auxiliary research technology, bioinformatics analysis has been widely used in various aspects of basic and clinical medical researches in recent years. At present, numbers of previous investigations have been performed based on the ovarian gene expression profiles available on NCBI Gene Expression Omnibus (GEO) database, and screened out a great many of differentially expressed genes (DEGs) that correlated with the development and progression of OC. For instance, Chen et al. (2020) screened out four hub genes as promising prognostic biomarkers for OC, followed by characterization of gene functions and mutual interactions employing bioinformatic analysis. Besides, Zhang et al. (2019) identified the potential prognostic and early detection biomarkers for OC by use of integrated bioinformatics approaches. Additionally, a previous study conducted utilizing bioinformatic analysis combined with experimental analysis proved that lncRNA FLJ33360 participated in the progression of OC through regulating miR-30b-3p (Yang et al., 2019). These lines of evidence testified that integrated bioinformatical approaches could make great contribution to the exploration for biomarkers and understanding of potential mechanism of the occurrence and development of OC.

In this research, DEGs were firstly screened out based on three OC-related microarray datasets (GSE14407, GSE18520, and GSE26712) obtained from the GEO database. Then, Gene Ontology (GO) analysis, protein–protein interaction (PPI) construction, and identification of hub genes were respectively conducted, followed by survival analysis. Finally, RP11-499E18.1, PAK2, and SOX2 were screened out as the target gene to carry out further investigation based on the results of bioinformatic analysis and previous published literatures. Thereafter, experimental analysis was further performed to reveal the underlying regulating mechanisms.

## Materials and Methods

### Microarray Data Analysis

In this study, three OC-related microarray datasets [GSE14407, GSE18520 (GPL570 platform), and GSE26712 (GPL96 platform)] were obtained from the GEO database^1^. The GSE14407 dataset contained 12 ovarian serous papillary carcinoma (OSPC) samples and 12 normal human ovarian surface epithelial (OSE) samples, while the GSE18520 had 53 advanced OC samples and 10 normal OSE samples. In addition, 185 primary OC samples and 10 normal OSE samples were included in the GSE26712 dataset (Table 1). Identification of the DEGs was performed with the help of an interactive online tool GEO2R^2^. The threshold for the DEGs was set as *p* < 0.05. Then, Venn analysis was conducted to identify the overlapping DEGs, and survival analysis was performed to screen the potential tumor suppressor genes.

**TABLE 1:** The information about the GEO datasets analyzed in this study.

**GEO accession**	**Platform**	**Sample size**
GSE14407	GPL570	24, OSE (*n* = 12), PSOC (*n* = 12)
GSE18520	GPL570	63, OSE (*n* = 10), advanced OC (*n* = 53)
GSE26712	GPL96	195, OSE (*n* = 10), primary OC (*n* = 185)

*GEO, Gene Expression Omnibus; OSE, ovarian surface epithelial; PSOC, papillary serous ovarian cancer; OC, ovarian cancer.*

### Gene Ontology Analysis

Gene ontology (GO) enrichment analysis of the commonly upregulated mRNAs was performed with the help of the Database for Annotation, Visualization, and Integrated Discovery (DAVID^3^). The top 10 pathways were selected to draw the bubble charts. *p* < 0.05 indicates a significant difference.

### catRAPID Database Analysis

catRAPID database^4^ is a tool for predicting the binding area of RNA and protein. In this study, catRAPID database was used to identify the interaction between the identified mRNA and proteins.

### Cell Lines, Ovarian Cancer Tissues, and Nude Mice

The OC cell lines (CaOV3, OVCAR3, SKOV3, A2780, HO-8910, and IOSE80) used in this study were obtained from the American Type Culture Collection (ATCC, Rockville, United States). CaOV3 and SKOV3 cells were respectively cultured in Dulbecco’s Modified Eagle’s Medium (DMEM, ATCC) and modified McCoy’s 5a Medium (ATCC), replenished with 10% fetal bovine serum (FBS, Gibco, Australia). OVCAR3, A2780, HO-8910, and IOSE80 cells were maintained in RPMI-1640 medium (ATCC) supplemented with 10% FBS. Cell cultivation was performed in a humidified, 5% CO_2_ and 37°C incubator, and the culture medium was replaced three times each week.

A total of eight pairs of OC tissues and its corresponding pericarcinomatous tissues were obtained from Hunan Cancer Hospital and the Affiliated Cancer Hospital of Xiangya School of Medicine (Changsha, China). One part was stored in a −80°C refrigerator (Harier, Qingdao, China), while another part was fixed in formalin (Aladdin, Shanghai, China) and then embedded in paraffin for subsequent immunohistochemical (IHC) staining. All patients who participated in this research were diagnosed as ovarian serous adenocarcinoma by two pathologists, and none of them received any radiotherapy or chemotherapy prior to surgery. Signed informed consent was obtained from each patient.

A total of 18 female BALB/c nude mice (aged 4–6 weeks) were acquired from the Experimental Animal Center of Hunan Cancer Hospital and the Affiliated Cancer Hospital of Xiangya School of Medicine (Changsha, China) and were divided into three groups (six mice/each group) in the following experiments. The animals were reared in a pathogen-free room under 45–50% humidity and 25–27°C temperature conditions. All procedures involved in this study had obtained the approval from the Animal Ethic Committee of the Hunan Cancer Hospital and the Affiliated Cancer Hospital of Xiangya School of Medicine Hospital.

CaOV3 cells were harvested in the exponential phase and then were digested into single-cell suspensions. After that, the nude mice, which had been reared under standard conditions for 1 week, were inoculated subcutaneously with 1 × 10^6^ cells in 0.2 ml of phosphate-buffered saline (PBS) at the right dorsal proximal upper limbs. The tumor size (width and length) was measured every 3 days for a total of six times after the tumor was visible to the naked eye. Tumor volume calculation was performed according to the formula listed below: tumor volume (V, mm^3^) = 0.5 × length × width. Additionally, the tumor tissues were collected, fixed in formalin, and embedded in paraffin for subsequent IHC staining to detect the expression of proliferation-related Ki67 and apoptosis-associated caspase-3.

### Quantitative Reverse-Transcription Polymerase Chain Reaction

RNA isolation was carried out from the collected tissues and cells utilizing TRIZOL reagent (TAKARA, Beijing, China). Genomic DNA was removed using RiboLock RNase Inhibitor and DNase I (Sangon Biotech, Shanghai, China). Quality control and concentration determination of the isolated RNA was fulfilled with the help of the spectrophotometer (HACH, Shanghai, China). RNA integrity was examined with 1% agarose gel electrophoresis. Then, reverse transcription was conducted with oligo(dT) primers utilizing Revertaid M Mulv Reverse Transcriptase (Thermo Fisher Scientific). Thereafter, qRT-PCR was carried out with the help of 2 × SYBR Green PCR Master mix (Thermo Fisher Scientific). Relative expression of target genes was calculated with the 2^–ΔΔCt^ method (Livak and Schmittgen, 2001). β-actin was set as the internal control. The primers used in this study were synthesized by Sangon Biotech and are listed in Table 2.

**TABLE 2:** The sequences of the primers used in qRT-PCR.

**Gene name**	**Primer sequences**
EGOT-F	5′-ACCGACTGTCCAACTAGCAA-3′
EGOT-R	5′-TTGTGTTTCCCTGTGCAGTG-3′
RP11-499E18.1-F	5′-AGCGTTGGGATTACAGGAGT-3′
RP11-499E18.1-R	5′-AGGACAGAAGCCAGAAGTTGA-3′
WT1-AS-F	5′-ACTCGTCTGTTCTGATGCCA-3′
WT1-AS-R	5′-ATGGGCCTACGTATCTGCTC-3′
HAND2-AS1-F	5′-TCCCCGAATCTGTAGTGTGG-3′
HAND2-AS1-R	5′-GAGTCACAGGCAGTCGTAGA-3′
PAK2-F	5′-TGAGCACACCATCCATGTTGG-3′
PAK2-R	5′-AGGTCTGTAGTAATCGAGCCC-3′
SOX2-F	5′-TACAGCATGTCCTACTCGCAG-3′
SOX2-R	5′-GAGGAAGAGGTAACCACAGGG-3′
β-actin-F	5′-ACCCTGAAGTACCCCATCGAG-3′
β-actin-R	5′-AGCACAGCCTGGATAGCAAC-3′
Primers U1-F	5′-GGGAGATACCATGATCACGAAGGT-3′
Primers U1-R	5′-CCACAAATTATGCAGTCGAGTTTCCC-3′

### Cell Transfection

The full-length complementary cDNA of human RP11-499E18.1 and PAK2 were synthesized by Sangon Biotech and cloned into pcDNA3.1 vector (TAKARA) to construct overexpression plasmids pcDNA-RP11-499E18.1 and pcDNA-PAK2. DNA Midiprep Kits (Thermo Fisher Scientific) were used for preparation of the overexpression plasmids. Meanwhile, the small interfering (si)RNA of RP11-499E18.1 (si-RP11-310-332, si-RP11-454-476 and si-RP11-589-611) and short harpin (sh)RNA of SOX2, as well as their corresponding negative control (NC) were synthesized to perform RNA knockdown. CaOV3 and SKOV3 cells were respectively plated in six-well plates (2 × 10^5^/well) and grown in a 37°C incubator overnight. Thereafter, the overexpression plasmids, pcDNA-RP11-499E18.1 and pcDNA-PAK2, were respectively transfected or co-transfected into the prepared cells. The knockdown sequences si-RP11-499E18.1 and shSOX2 were respectively transfected into cells. Besides, co-transfection of pcDNA-PAK2 and shSOX2 were also performed. Cell transfections were performed with the help of Lipofectamine 3000 (Invitrogen, California, United States). Cells were harvested 48 h post transfection to conduct further experiments. The sequences of the plasmids, siRNAs, and shRNAs are detailed in Table 3.

**TABLE 3:** The sequences of siRNAs and shRNAs used in cell transfection.

**Name**	**Sequences**
si-RP11-310-332	5′-UCCAUAUCCUCUUAACCAGGA CUGGUUAAGAGGAUAUGGAUA-3′
si-RP11-454-476	5′-UCCUUUUAUCUUUGUCUUCAU GAAGACAAAGAUAAAAGGAAC-3′
si-RP11-589-611	5′-UAGGAUAUGGUAAACACUGUU CAGUGUUUACCAUAUCCUAAU-3′
si-PAK2	5′-AGAAGGAACUGAUCAUUAA-3′
si’-PAK2	5′-GAAACUGGCCAAACCGUUAUU-3′
shSOX2-1	5′-CGAGATAAACATGGCAATCAA-3′
shSOX2-2	5′-GTACAGTATTTATCGAGATAA-3′

### Cell Proliferation

After transfection, (3-(4,5-Dimethylthiazol-2-yl)-2,5-diphenyltetrazoliumbromide) MTT assay was carried out to assess cell proliferation. Briefly, 100 μl of the transfected CaOV3 and SKOV3 cells was resuspended and seeded into 96-well plates at a concentration of 1 × 10^4^ cells per well. Then, 50 μl of MTT reagent that was dissolved in PBS was respectively provided into each well at 0, 12, 24, 48, and 72 h after transfection, and the mixtures were maintained in a 37°C incubator for another 4 h. Thereafter, 150 μl of dimethyl sulfoxide (DMSO) was supplied into each well to dissolve the formazan. The optical density (OD) at 570 nm was measured utilizing a microplate reader (Bio-Rad, Hercules, CA, United States). Cell proliferation ratio (%) was calculated according to the formula listed below: Cell proliferation ratio (%) = OD_experimental group_/OD_NC_ × 100%.

After transfection, CaOV3 and SKOV3 cells were resuspended and plated into 6-well plates at a concentration of 1,000 cells per well. Then, the plates were maintained in a humidified incubator under 5% CO_2_ and 37°C conditions for 2–3 weeks. Colony fixation and staining were respectively conducted utilizing methanol and 0.1% crystal violet (Sangon Biotech). Finally, the images of the colonies were photographed with a microscope (Olympus, Tokyo, Japan).

### Transwell Assay

After transfection, the invasive capacity of CaOV3 and SKOV3 cells was determined using transwell assay. Briefly, the Matrigel chamber (Corning, New York, United States) used in this study was coated with 50 μl Matrigel (1:8) in advance. CaOV3 and SKOV3 cells were suspended in serum-free medium at a concentration of 5 × 10^4^/ml. Then, 200 μl of the suspended cells was added to the upper chamber, and 600 μl of complete medium was provided to the lower chamber. After maintaining for 48 h in a 5% CO_2_ and 37°C incubator, the uninvaded cells on the upper chamber were wiped off, while the successfully invaded cells on the lower chamber were fixed with 95% ethanol (Aladdin) for 20 min and stained with hematoxylin (Sangon Biotech) for 10 min. Finally, the images of the invaded cells were photographed with a microscope (Olympus).

### Immunohistochemical

The prepared tissues were respectively sliced into 3-μm-thick sections, and baked in a 60–65°C incubator for 4 h. Then, the slices were dewaxed in xylene, rehydrated in grade ethanol, and rinsed with 1× PBS for three times. Afterward, the slices were put into the citrate buffer (10 mmol/L, pH 6.0) and heated to 121°C in an autoclave for 20 min. The endogenous peroxidase was blocked in 50 μl of 5% H_2_O_2_ for 10 min at room temperature (RT). Subsequently, incubation of primary antibodies, including anti-PAK2 (ab76293, Abcam, Cambridge, United Kingdom), anti-SOX2 (#2748, Cell Signal Technology, Boston, United States), anti-Caspase3 (ab4051, Abcam), and anti-Ki67 (ab15580, Abcam), was performed at 4°C overnight. After rinsing, each slice was incubated with 50 μl of HRP-labeled secondary antibody (ab6721, Abcam) for 10 min at RT. Then, 50 μl of streptomyces anti-biotin-peroxidase solution was provided to each slice and reacted for 10 min. After rinsing, the slices were incubated with 3,39-diaminobenzidine tetrahydrochloride (DAB) reagent for approximately 10 min to develop color. Then, the reaction was terminated, and hematoxylin re-staining was performed, followed by dehydration and transparency, and neutral resin mounting. For the NC group, the slices were incubated with PBS instead of primary antibodies. Each slice was respectively photographed at 200 and 400× magnification with the help of a microscope (Olympus).

### Immunofluorescence

CaOV3 and SKOV3 cells were respectively seeded into six-well plates with the autoclaved cover glasses placed at the bottom of the wells. After cells grown to 50% confluence on the coverslips, PBS rinsing was performed, followed by 4% paraformaldehyde (PFA) fixation at RT for 10 min. After washing, cells were permeated with PBS, which contains 0.5% Triton X-100 for 20 min and blocked with 3% bovine serum albumin (BSA, Thermo Fisher Scientific) for 1 h at RT. After rinsing with PBS, primary antibody anti-SOX2 (#92186, Cell Signal Technology) incubation was carried out with the cells attached on the coverslips at 4°C overnight. Alexa Fluor^®^ 488-conjugated secondary antibody (ab150077, Abcam) incubation was conducted at RT for 1 h. Then, the cell nuclei were counterstained with 4-6-diamidino-2-phenylindole (DAPI) reagent (Sangon Biotech) for 10 min and the slices were mounted. Finally, images were observed and photographed using a confocal microscope (Zeiss, Jena, Germany).

### Fluorescence *in situ* Hybridization

The RP11-499E18.1 and U6 probes were both purchased from RiboBio (Guangzhou, China). CaOV3 and SKOV3 cells were respectively seeded into 24-well plates (approximately 6 × 10^4^/well) with the autoclaved cover glasses placed at the bottom of the wells. After the cells were grown to 60–70% confluence on the coverslips, 4% PFA fixation was performed at RT for 10 min, followed by PBS rinsing. Afterward, cells were permeabilized with 1 ml of pre-cooled PBS containing 0.5% Triton X-100 at 4°C for 5 min. Then, the supernatant was discarded. After that, 200 μl of pre-hybridization reagent was provided and reacted for 30 min at 4°C. Thereafter, the pre-hybridization reagent was removed. The hybridization solution containing RP11-499E18.1 or U6 probes were respectively added under dark conditions, and the solution was maintained at 37°C overnight. Then, 4 × SSC (containing 0.1% Tween-20) rinsing was performed to reduce the background signal, followed by 2 × SSC, 1 × SSC, and PBS rinsing under dark conditions. Subsequently, DAPI reagent was added and reacted for 10 min to counterstain cell nuclei. After rinsing, the slices were mounted and subjected to fluorescent signal detection employing a confocal microscopy (Zeiss).

### Western Blot

Protein extraction was performed by use of RIPA lysis buffer (Beyotime, Shanghai, China), in the presence of protease inhibitors (Thermo Fisher Scientific). Quantification of the extracted proteins was fulfilled employing a Nanodrop 2000 system (Thermo Scientific). Afterward, protein separation was carried out utilizing SDS-PAGE. Subsequently, the target proteins were transferred onto the nitrocellulose (NC) membrane, and blocked in 5% BSA. Thereafter, incubation of primary antibodies, including anti-PAK2 (ab76293, Abcam), anti-SOX2 (#2748, Cell Signal Technology), anti-p-SOX2 (#92186, Cell Signal Technology), anti-E-cadherin (ab40772, Abcam), anti-Vimentin (ab137321, Abcam), and anti-β-actin (66009-1-Ig, Ptgcn, IL, United States), was conducted at 4°C overnight. After that, the target proteins were incubated with HRP-labeled secondary antibody (ab6721, Abcam) at RT for 1 h. Finally, the signals were developed using ECL reagent and detected with the help of a LAS-3000 (FUJIFILM, Tokyo, Japan) system.

### RNA Pulldown Assay

The biotin-labeled full-length RP11-499E18.1 and antisense RP11-499E18.1 were synthesized using Biotin RNA Labeling Mix (Roche, Basel, Switzerland) and T7 RNA polymerase (Promega, Wisconsin, United States), in the presence of RNase-free DNase I (Thermo Fisher Scientific). Then, the products were purified with the help of RNeasy Mini Kit (Qiagen, MD, United States). Afterward, the proteins exacted from CaOV3 and SKOV3 cells were respectively incubated with the biotinylated RNA at RT for 4 h. Thereafter, the streptavidin magnetic beads (Thermo Fisher Scientific) were provided and the mixtures were maintained at 4°C overnight. After that, the unbounded proteins were removed, while the bounded proteins were eluted and further analyzed by electrophoresis, silver staining, mass spectrometry (MS), and Western blot. The oligonucleotide sequences used for RNA pulldown are listed in Table 4.

**TABLE 4:** The oligonucleotide sequences used for RNA pulldown assay.

**Name**	**Sequences**
RP11-499E18.1-sense-F	5′-TAATACGACTCACTATAGGGAGACCTCAGCGTCCGGAG TAGCTA-3′
RP11-499E18.1-sense-R	5′-TTGCAAGTTAGAGCACTATATT-3′
RP11-499E18.1-antisense-F	5′-CCTCAGCGTCCGGAGTAGCTA-3′
RP11-499E18.1-antisense-R	5′-TAATACGACTCACTATAGGGAGATTGCAAGTTAGAGCAC TATATT-3′

### RNA Immunoprecipitation Assay

RNA immunoprecipitation (RIP) was carried out utilizing EZ-Magna RIP Kit (Millipore, United States) following the manufacturer’s instructions. Briefly, CaOV3 and SKOV3 cells were lysed in RIP lysis buffer, and then the cell lysates were incubated with RIP buffer, with magnetic beads conjugated with AGO2 antibody or NC mouse IgG with rotation at 4°C overnight. After centrifugation, the beads were collected and rinsed by RIP Wash Buffer. Thereafter, the complexes were incubated with proteinase K buffer to remove proteins. After that, isolation and purification of the immunoprecipitated RNA was performed. Further analysis was conducted utilizing qRT-PCR.

### Co-immunoprecipitation

For co-immunoprecipitation (co-IP), both of the untransfected and transfected CaOV3 and SKOV3 cells were incubated with RIPA lysis buffer (Beyotime), in the presence of protease inhibitors (Thermo Fisher Scientific). Afterward, the supernatants of cell lysates were collected by centrifugation and then were respectively incubated with primary antibodies, including PAK2 antibody and SOX2 antibody, or control IgG at 4°C overnight. After that, the Protein A/G PLUS-Agarose beads (Santa Cruz Biotechnology) were provided and the mixtures were maintained at 4°C for 2 h. Thereafter, the beads were isolated and rinsed with lysis buffer, and finally analyzed by Western blot.

### Statistical Analysis

Each experiment possesses three replications. Data analysis was completed employing GraphPad Prism (GraphPad software, CA, United States). Data were expressed as mean ± standard deviation (SD). The association between RP11-499E18.1, PAK2, and SOX2 expression with the overall survival (OS) was determined with the Kaplan–Meier survival analysis using the log-rank test. Comparison between two groups and among multi-groups was respectively conducted utilizing Student’s *t*-test and one-way ANOVA followed by Tukey’s *post hoc* test. *p* < 0.05 represents statistically significant.

## Results

### RP11-49E18.1 Was Verified to Be a Potential Tumor Suppressor Gene of Ovarian Cancer

**FIGURE 1:**
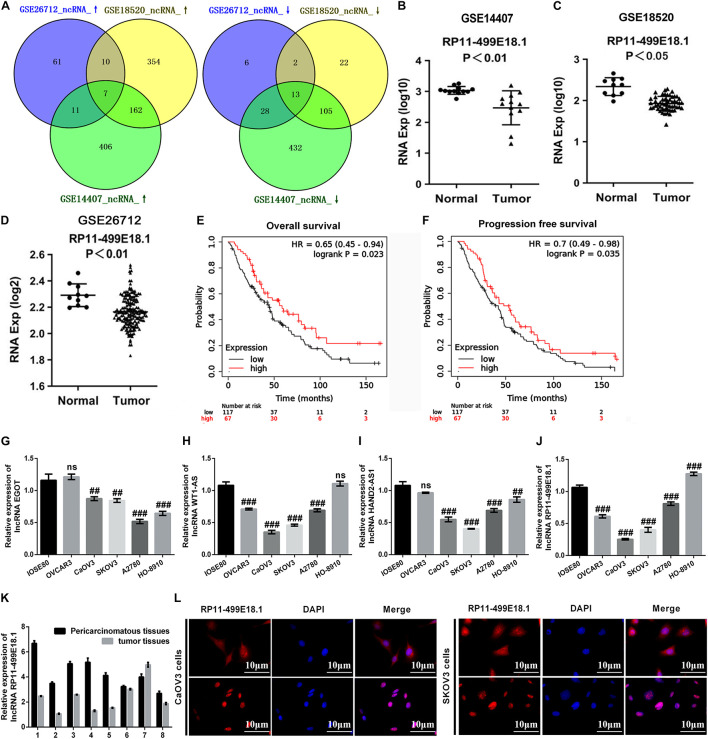
RP11-49E18.1 was verified to be a potential tumor suppressor gene of OC. **(A)** Venn diagram analysis respectively identified 7 upregulated and 13 downregulated DEGs. **(B–D)** RP11-499E18.1 was distinctly downregulated in OC tissues. **(E,F)** Low expression of RP11-499E18.1 was closely associated with poor prognosis of OC patients. **(G–J)** RP11-499E18.1 was the lowest expressed gene in a series of OC cells, especially in CaOV3 and SKOV3 cells. **(K)** RP11-499E18.1 expression in OC tissues was notably lower than in pericarcinomatous tissues. **(L)** RP11-499E18.1 exists in cells in the form of plasma–nucleus coexistence. DEGs, differentially expressed genes; OC, ovarian cancer. ^##^*p* < 0.01, ^###^*p* < 0.001, ns, no significance.

In this study, a variety of bioinformatics approaches were employed to identify the genes that played crucial roles in the regulation of OC. Analysis of GSE14407, GSE18520, and GSE26712 respectively identified 1,164 (586 upregulated, 578 downregulated), 675 (533 upregulated, 142 downregulated), and 138 (89 upregulated, 49 downregulated) DEGs. Venn analysis discovered that there were a total of 20 consistently expressed genes, of which 7 were upregulated, while 13 were downregulated (Figure 1A). Based on these results, survival analysis was further performed to evaluate the relationship between these genes and survival prognosis. Outcomes showed that the four downregulated lncRNAs, including EGOT, WT1-AS, HAND2-AS1, and RP11-499E18.1, exerted typical low expression and poor prognosis, which indicates that these four lncRNAs might be the potential tumor suppressor genes of OC. As shown in Figures 1B–D, RP11-499E18.1 was significantly downregulated in different types of OC (*p* < 0.01 or *p* < 0.05). Besides, survival analysis results showed that low expression of RP11-499E18.1 was closely associated with poor prognosis of OC patients (Figures 1E,F, *p* < 0.05), in which the RP11-499E18.1 high expression group accounts for 40% according to the cutoff values. Afterward, we detected the expression of EGOT, WT1-AS, HAND2-AS1, and RP11-499E18.1 in a series of OC cells. Result shown in Figures 1G–J showed that RP11-499E18.1 was the lowest expressed gene, especially in CaOV3 and SKOV3 cells (*p* < 0.01 or *p* < 0.001). Subsequently, the expression of RP11-499E18.1 in eight pairs of clinical OC tissues was also detected. Results showed that its expression in OC tissues was notably lower than in pericarcinomatous tissues (Figure 1K). In addition, the location of RP11-499E18.1 in OC cells was further determined by FISH, and outcomes demonstrated that RP11-499E18.1 exists in cells in the form of plasma–nucleus coexistence (Figure 1L). Therefore, RP11-499E18.1 was chosen to conduct further research in this study.

### PAK2 Was Predicted to Be an Oncogene of Ovarian Cancer

**FIGURE 2:**
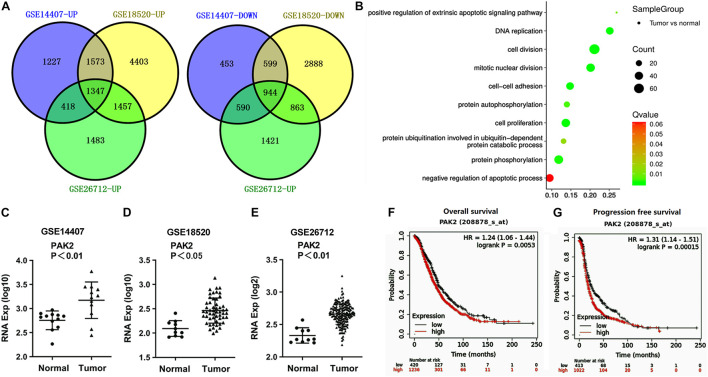
PAK2 was predicted to be an oncogene of OC. **(A)** Venn diagram analysis respectively identified 1,347 upregulated and 944 downregulated differentially expressed mRNAs. **(B)** The top 10 pathways obtained by GO term analysis. **(C–E)** PAK2 expression was distinctly upregulated in OC tissues. **(F,G)** High expression of PAK2 was closely associated with poor prognosis of OC patients. OC, ovarian cancer; GO, Gene Ontology; PAK2, P21 (RAC1) Activated Kinase 2.

In order to explore how RP11-499E18.1 exerts its regulatory roles in OC, we further analyzed these datasets to figure out the crucial mRNAs participated in the regulation of OC. There were respectively 7,132 (4,546 upregulated, 2,586 downregulated), 14,074 (8,780 upregulated, 5,294 downregulated), and 8,523 (4,705 upregulated, 3,818 downregulated) differentially expressed mRNAs screened out. Venn diagram exhibited that there were a total of 2,291 consistently expressed mRNAs, namely, 1,347 upregulated and 944 downregulated genes (Figure 2A). Considering that the upregulated mRNAs were much more than downregulated mRNAs in OC, GO enrichment analysis was performed among the upregulated mRNAs. Results showed that 2 of the top 10 pathways were related to protein phosphorylation (Figure 2B). Besides, PAK2 was found to exist in several typical GO pathways mentioned above (Table 5), indicating that PAK2 might be the functional protein of RP11-499E18.1. Thereafter, PAK2 expression in OC tissues and the association between PAK2 expression and prognosis of OC patients were further analyzed. Results shown in Figures 2C–E demonstrated that PAK2 was notably upregulated in different types of OC (*p* < 0.01 or *p* < 0.05). Besides, survival analysis revealed that there was a close association between high expression of PAK2 and poor prognosis of OC patients (Figures 2F,G, *p* < 0.01), among which PAK2 high expression groups account for 75% according to the cutoff values. These results indicated that PAK2 might exert a pro-cancer efficacy in OC.

**TABLE 5:** Gene ontology (GO) analysis of five typical pathways associated with ovarian cancer (OC).

**Term/gene function**	**Genes**
GO:0006468 ∼protein phosphorylation	NUAK2, FASTK, PASK, PKMYT1, RPS6KB2, AURKA, AURKB, PRKY, CCNE1, ACVR1B, ADCK2, CSNK2A1, PAK2, TLK1, CDK16, CSK, LIMK1, PHKG2, PRKCI, PKN1, MINK1, PBK, SRPK1, GAK, CCND1, MAST2, HIPK2, BUB1B, WNT11, EIF2AK2, LRRK1, KALRN, NEK2, MAP4K1, MAPKAPK2, BUB1, CAMK2B, DYRK2, STK19, RUNX3, AATK, CSNK1A1, TAF1, TRIO, BIRC5, ILF3, IKBKE, CSNK1E, GSK3A, GRK6, TSSK2, JAK3, CIT, IKBKB
GO:0098609 ∼cell–cell adhesion	ALDOA, YWHAZ, SEPT2, ZC3HAV1, RANGAP1, SFN, TAGLN2, PKM, EPCAM, PAK6, BZW2, PAK2, DDX3X, CC2D1A, CLINT1, TES, GOLGA2, HIST1H3J, STX5, MYO6, BAIAP2, TRIM29, CBL, DOCK9, PFKP, LYPLA2, MICALL1, CORO1B, EPB41L1, CCNB2, HIST1H3B, HIST1H3E, ERC1, HIST1H3F, DBN1, HIST1H3G, HIST1H3H, PUF60, HIST1H3I, SEPT9
GO:0046777 ∼protein autophosphorylation	FGFR2, TAF1, FLT1, NEK2, PASK, MAP4K1, TTK, MINK1, AURKA, CAD, MAPKAPK2, AURKB, EPHB3, PRKX, ACVR1B, DDR1, PTK2, PAK2, MAP3K10, TSSK2, CAMK2B, EIF2AK2, CSK, MELK
GO:2001238 ∼positive regulation of extrinsic apoptotic signaling pathway	BID, LTBR, PAK2, TNFRSF12A, PML, RBCK1, WWOX
GO:0043066 ∼negative regulation of apoptotic process	STIL, CLDN7, PPARD, YWHAZ, HTATIP2, MCL1, NUAK2, AURKA, BCL2L1, SOX9, ADORA1, EPCAM, PTK2, PAK2, DDX3X, ITCH, ARHGDIA, DHCR24, KIF14, CDK1, PRAME, SOCS3, SMAD6, CBL, TP53, PRKCI, BIRC5, HMGA2, PPIF, ATF5, PA2G4, HSP90B1, MSX1, MAD2L1, UCP2, VEGFA, TFAP2A, WNT11, IKBKB, EIF2AK2, CLEC5A, GSTP1, BARD1
*p* ≤ 0.01	

### Validation of the Interactions Among RP11-499E18.1, PAK2, and SOX2

**FIGURE 3:**
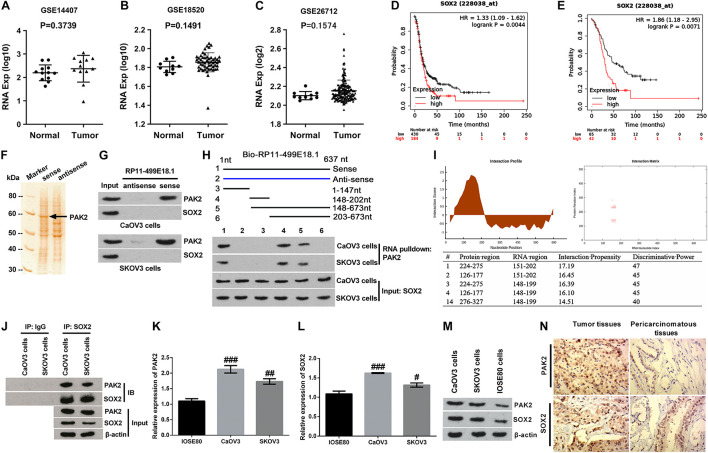
Validation of the interactions among RP11-499E18.1, PAK2, and SOX2. **(A–C)** There was no significant difference of SOX2 expression between normal tissues and OC tissues. **(D,E)** High expression of SOX2 was closely associated with poor prognosis of OC patients. **(F)** The band located at 60 kDa was identified as PAK2. **(G)** RNA pulldown assay affirmed that there was an interaction between RP11-499E18.1 and PAK2. **(H,I)** Domain mapping assay and catRAPID database analysis confirmed that there was a strong binding effect between the 150–200 bp region of lncRNA RP11-49E18.1 and the 126–177/245–275 amino acid region of PAK2. **(J)** There was an interaction between PAK2 and SOX2 in CaOV3 and SKOV3 cells. **(K–M)** The expression of PAK2 and SOX2 in CaOV3 and SKOV3 cells was notably higher than in IOSE80 cells. **(N)** The expression of PAK2 and SOX2 in OC tissues was much higher than in pericarcinomatous tissues. OC, ovarian cancer; PAK2, P21 (RAC1) activated kinase 2; SOX2, SRY-box transcription factor 2. ^#^*p* < 0.05, ^##^*p* < 0.01, ^###^*p* < 0.001.

Numbers of previous investigations have clarified the interaction between PAK2 and SOX2 in various human diseases (Huttlin et al., 2015, 2017). Therefore, relative expression of SOX2 in OC tissues and the correlation between SOX2 expression and the prognosis of OC patients were also analyzed. Outcomes displayed in Figures 3A–C showed that there was no significant change of SOX2 expression between OC tissues and normal tissues. Besides, survival analysis revealed that SOX2 was a poor prognostic factor (Figures 3D,E), in which the SOX2 high expression group accounts for 30% according to cutoff values. Combined with the results of PAK2 GO enrichment analysis, these results indicated that PAK2 might function through regulating the phosphorylation level of SOX2, which could further influence gene transcription.

Based on the aforementioned results, we deduced that RP11-499E18.1 might exert its anti-cancer efficacy in OC *via* regulation of the RP11-499E18.1–PAK2–SOX2 axis. Therefore, RNA pulldown assay was carried out to explore whether there exist an association among RP11-499E18.1, PAK2, and SOX2. MS analysis revealed that the protein with a molecular weight close to 60 kDa in the electrophoresis was PAK2 (Figure 3F). In addition, results displayed in Figure 3G confirmed that RP11-499E18.1 could directly bind to PAK2 in both CaOV3 and SKOV3 cells, while there was no interaction between RP11-499E18.1 and SOX2. After that, a series of biotin-labeled RP11-499E18.1 fragments were constructed to determine the specific and effective binding region of RP11-499E18.1 when binding to PAK2. Results showed that the 148–202 bp region of RP11-499E18.1 is the possible binding region of PAK2 (Figure 3H). Meanwhile, catRAPID database analysis revealed that there was a strong binding effect between the 150–200 bp region of lncRNA RP11-49E18.1 and the 126–177/245–275 amino acid region of PAK2 (Figure 3I). Thereafter, Co-IP assay was conducted to confirm whether there exists an interaction between PAK2 and SOX2. Results verified that there was an association between PAK2 and SOX2 in CaOV3 and SKOV3 cells (Figure 3J). Then, the expression of PAK2 and SOX2 in OC cells and tissues was respectively testified. qRT-PCR and Western blot results displayed that both PAK2 and SOX2 in CaOV3 and SKOV3 cells was notably higher than their expression in IOSE80 cells (Figures 3K–M, *p* < 0.05 or *p* < 0.01 or *p* < 0.001). IHC results demonstrated that the expression of PAK2 and SOX2 in OC tissues was much higher than in pericarcinomatous tissues (Figure 3N). These results indicated that RP11-49E18.1 might perform its tumor-suppressive roles through influencing its interaction with PAK2 and the interaction between PAK2 and SOX2.

### RP11-499E18.1 Overexpression Obviously Suppressed Cell Proliferation, Migration, Colony Formation, and Epithelial–Mesenchymal Transition Transformation

**FIGURE 4:**
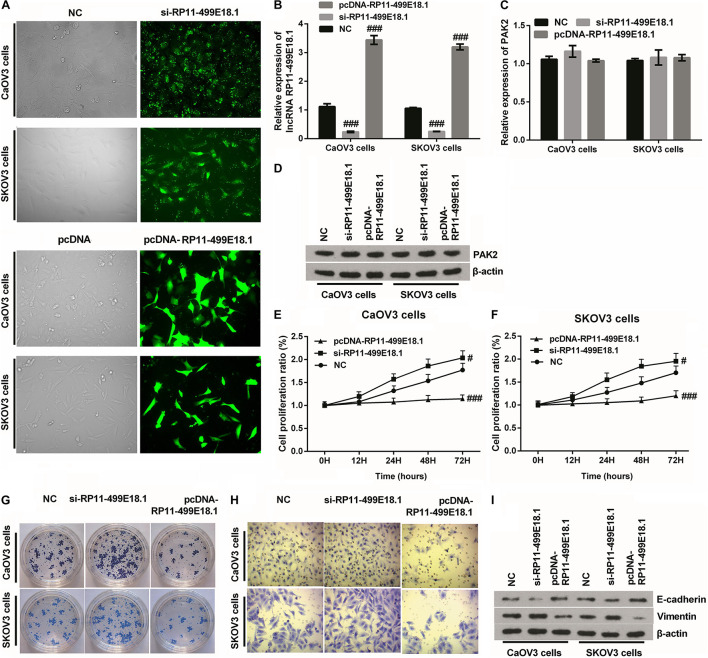
RP11-499E18.1 overexpression obviously suppressed cell proliferation, migration, colony formation, and EMT transformation. The sequences of NC and si-RP11-499E18.1 were respectively transfected into CaOV3 and SKOV3 cells to silence RP11-499E18.1 expression. The plasmids pcDNA 3.1 and pcDNA-RP11-499E18.1 were respectively transfected into CaOV3 and SKOV3 cells to overexpress RP11-499E18.1. **(A,B)** The transfection efficiency was high, and RP11-499E18.1 was successfully silenced and overexpressed in CaOV3 and SKOV3 cells. **(C,D)** There was no obvious influence of RP11-499E18.1 knockdown or overexpression on the expression of PAK2. **(E–H)** The proliferation, colony formation, and migration of CaOV3 and SKOV3 cells were distinctly repressed by RP11-499E18.1 overexpression, while notably promoted by RP11-499E18.1 knockdown. **(I)** The expression of EMT-related E-cadherin was distinctly increased, while Vimentin was notably decreased by RP11-499E18.1 overexpression. RP11-499E18.1 knockdown performed the opposite effects. NC, negative control; si, small interfering; PAK2, P21 (RAC1) activated kinase 2; EMT, epithelial–mesenchymal transition. ^#^*p* < 0.05, ^###^*p* < 0.001.

After transfection, the transfection efficiency was respectively testified employing qRT-PCR and fluorescence microscope. Outcomes verified that the expression of RP11-499E18.1 was successfully knocked down or overexpressed after cell transfection (Figures 4A,B and Supplementary Figure 1, *p* < 0.01 or *p* < 0.001), and the knockdown efficiency was especially high when cells were transfected with si-RP11-454-476. Afterward, the expression of PAK2 was detected, and outcomes demonstrated that overexpression or knockdown of RP11-499E18.1 had no significant influence on the expression of PAK2 on both mRNA and protein levels (Figures 4C,D). Thereafter, cell proliferation, migration, and colony formation, as well as the expression of EMT transformation-related factors (E-cadherin and Vimentin) were tested in the transfected cells. Outcomes demonstrated that overexpression of RP11-499E18.1 obviously suppressed the proliferation, migration, and colony formation of both CaOV3 and SKOV3 cells (Figures 4E–H, *p* < 0.01 or *p* < 0.001). Besides, the expression of EMT transformation-related E-cadherin was notably augmented, while the expression of Vimentin was obviously decreased by RP11-499E18.1 overexpression in both CaOV3 and SKOV3 cells (Figure 4I). However, RP11-499E18.1 knockdown exerted the opposite effects as RP11-499E18.1 overexpression is performed (Figures 4E–I).

### PAK2 Upregulation Notably Counteracted RP11-499E18.1 Overexpression-Triggered Tumor-Suppressing Effects

**FIGURE 5:**
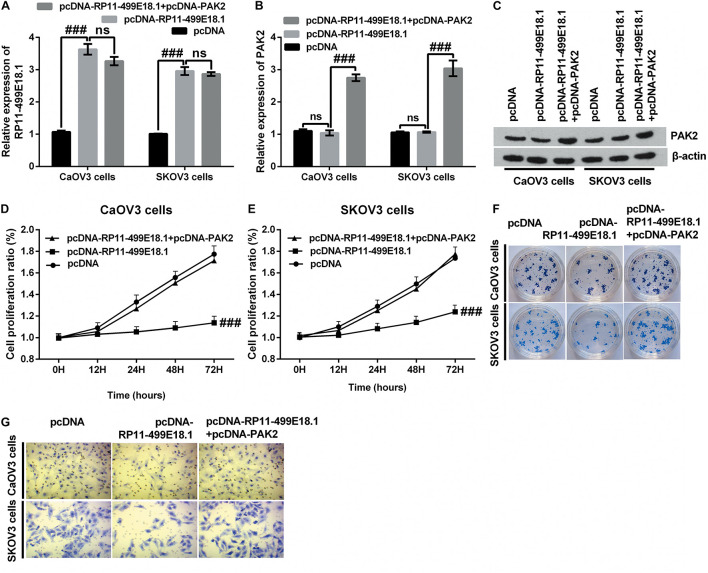
PAK2 upregulation notably counteracted RP11-499E18.1 overexpression-triggered tumor-suppressing effects. The plasmids pcDNA3.1, pcDNA-RP11-499E18.1, and pcDNA-RP11-499E18.1 + pcDNA-PAK2 were respectively transfected into CaOV3 and SKOV3 cells. **(A)** RP11-499E18.1 was successfully overexpressed in CaOV3 and SKOV3 cells. **(B,C)** PAK2 was successfully overexpressed in CaOV3 and SKOV3 cells. **(D–G)** PAK2 overexpression distinctly counteracted RP11-499E18.1 overexpression-triggered suppressing effects on the proliferation, colony formation, and migration of CaOV3 and SKOV3 cells. PAK2, P21 (RAC1) activated kinase 2. ^###^*p* < 0.001, ns, no significance.

Previous RNA pulldown assay verified that there was an interaction between RP11-49E18.1 and PAK2. Based on this result, we speculated that RP11-49E18.1 might perform its tumor-suppressive roles through interacting with PAK2. Therefore, we respectively co-overexpressed RP11-49E18.1 and PAK2 in CaOV3, SKOV3, and OVCAR3 cells. Results displayed that co-transfection with pcDNA-RP11-49E18.1 and pcDNA-PAK2 had no significant influence on the expression of RP11-49E18.1 (Figure 5A and Supplementary Figure 2), while it had distinctly increased the expression of PAK2 compared with pcDNA-RP11-49E18.1 transfected cells (Figures 5B,C, both *p* < 0.01). Following experiments further verified that co-overexpression of RP11-49E18.1 and PAK2 notably counteracted RP11-499E18.1 overexpression-triggered suppressing effects on the proliferation, migration, and colony formation of CaOV3 and SKOV3 cells (Figures 5D–G, *p* < 0.01 or *p* < 0.001). These outcomes indicated that PAK2 might perform a tumor-promoting efficacy in OC, which is consistent with the prognosis analysis results.

### Knockdown of SOX2 Notably Reversed PAK2 Overexpression-Triggered Tumor-Promoting Effects

**FIGURE 6:**
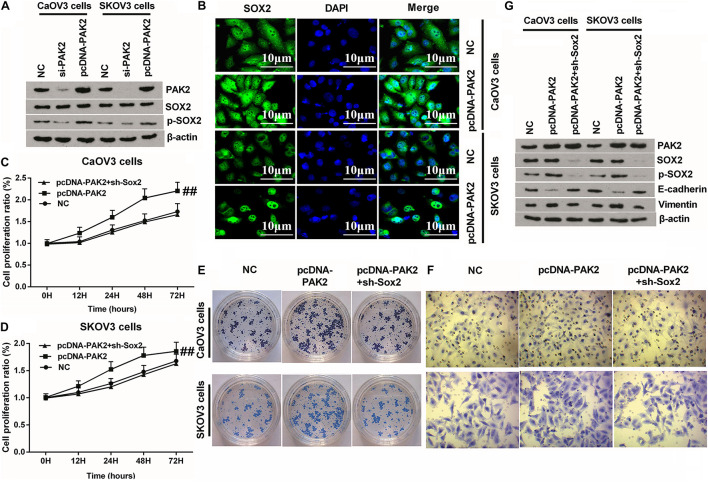
Knockdown of SOX2 notably reversed PAK2 overexpression-triggered tumor-promoting effects. **(A)** PAK2 overexpression notably have no notable influence on the expression of SOX2, but obviously increased the expression of p-SOX2. **(B)** PAK2 overexpression promoted the nuclear translocation of SOX2. **(C–F)** Knockdown of SOX2 obviously reversed PAK2 overexpression-induced augmenting effects on the proliferation, colony formation, and migration of CaOV3 and SKOV3 cells. **(G)** PAK2 overexpression obviously decreased the expression of EMT-associated E-cadherin, and increased the expression of Vimentin. Knockdown of SOX2 notably counteracted PAK2 overexpression-induced effects. PAK2, P21 (RAC1) activated kinase 2; SOX2, SRY-box transcription factor 2; EMT, epithelial–mesenchymal transition. ^##^*p* < 0.01.

Considering that PAK2 is a member of the serine/threonine kinases family, bioinformatic analysis revealed that there were two pathways related to protein phosphorylation. Besides, previous investigation verified that the phosphorylated SOX2 could be transferred into the nucleus and promote gene transcription (Ravindran Menon et al., 2018; Wang Z. et al., 2019). Therefore, we detected the expression of p-SOX2 in OC cells. Western blot results displayed that there was no obvious change between the expression of SOX2 in both PAK2 knockdown or overexpressed cells. However, p-SOX2 expression was distinctly decreased by PAK2 knockdown, while notably upregulated by PAK2 overexpression (Figure 6A and Supplementary Figure 3). Therefore, we deduced that PAK2 overexpression might have promoted the nuclear translocation of SOX2, and this speculation was subsequently testified by the IF assay results (Figure 6B). Afterward, NC, pcDNA-PAK2, and pcDNA-PAK2 plus sh-SOX2 transfection was respectively conducted in CaOV3 and SKOV3 cells to explore the underlying regulating mechanism. Detection of cell phenotype confirmed that PAK2 overexpression obviously promoted the proliferation, migration, and colony formation of CaOV3 and SKOV3 cells (Figures 6C–F, *p* < 0.01 or *p* < 0.001). Besides, the expression of p-SOX2 and EMT-related Vimentin was both notably augmented, while E-cadherin expression was significantly reduced by PAK2 overexpression. Meanwhile, no significant change of SOX2 expression was observed (Figure 6G). More importantly, these tumor-promoting effects triggered by PAK2 overexpression were distinctly reversed by SOX2 knockdown (Figures 6C–G). Combination of these results indicated that PAK2 overexpression might perform its tumor-promoting efficacy through increasing the phosphorylation of SOX2.

### RP11-499E18.1 Overexpression Reduced the Interaction Between PAK2 and SOX2 and Increased the Binding of RP11-499E18.1 by PAK2

**FIGURE 7:**
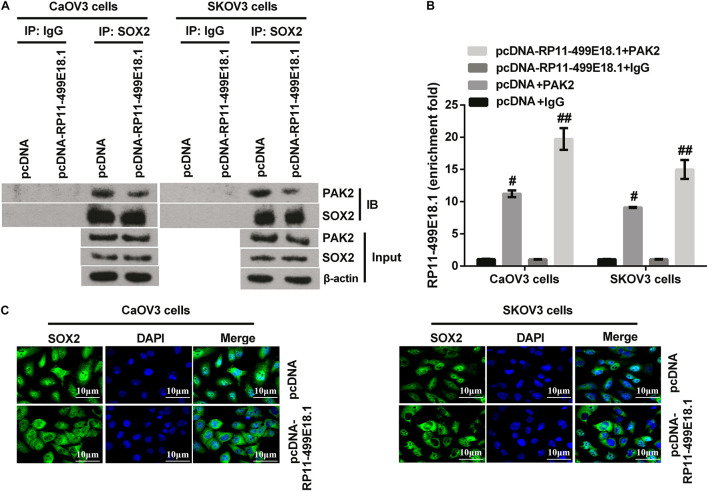
RP11-499E18.1 overexpression reduced the interaction between PAK2 and SOX2, and increased the binding of RP11-499E18.1 by PAK2. **(A)** Co-IP assay confirmed that RP11-499E18.1 overexpression remarkably reduced the interaction between PAK2 and SOX2. **(B)** RIP assay affirmed that RP11-499E18.1 overexpression notably increased the binding of RP11-499E18.1 by PAK2. **(C)** Overexpression of RP11-499E18.1 remarkably decreased the nuclear translocation of SOX2 in CaOV3 and SKOV3 cells. Co-IP, co-immunoprecipitation; RIP, RNA immunoprecipitation; PAK2, P21 (RAC1) activated kinase 2; SOX2, SRY-box transcription factor 2. ^#^*p* < 0.05, ^##^*p* < 0.01.

For further disclosing the regulating mechanism among RP11-499E18.1, PAK2, and SOX2, co-IP and RIP assay were performed in RP11-499E18.1-overexpressing cells. Results of co-IP assay revealed that RP11-499E18.1 overexpression markedly reduced the interaction between PAK2 and SOX2 compared with the NC group (Figure 7A). Besides, outcomes of RIP assay disclosed that RP11-499E18.1 overexpression distinctly increased the binding of RP11-499E18.1 by PAK2 (Figure 7B, *p* < 0.05 or *p* < 0.01). In addition, detection of the location of SOX2 in RP11-499E18.1-overexpressed CaOV3 and SKOV3 cells was carried out. Outcomes displayed that RP11-499E18.1 overexpression obviously reduced the nuclear translocation of SOX2, and SOX2 is more localized in the cytoplasm compared with pcDNA transfected cells (Figure 7C). These outcomes manifested that RP11-499E18.1 overexpression might carry out its tumor-suppressing efficacy through decreasing the interaction between PAK2 and SOX2, and thereby lessen the nuclear translocation of SOX2.

### PAK2 Upregulation Reversed RP11-499E18.1 Overexpression-Induced Facilitating Effects on Tumor Formation

**FIGURE 8:**
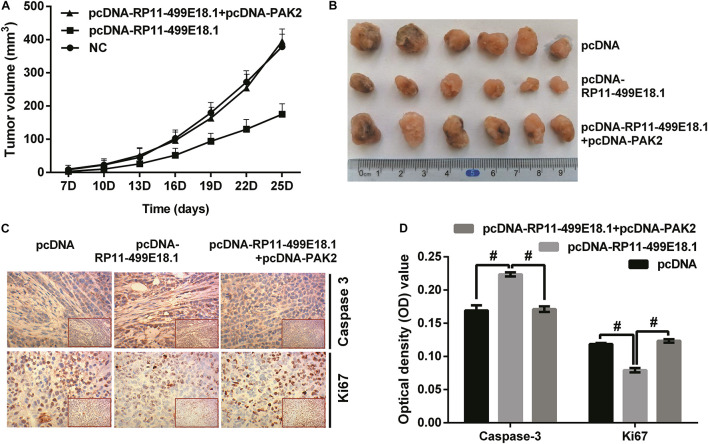
PAK2 upregulation reversed RP11-499E18.1 overexpression-induced facilitating effects on tumor formation. The plasmids pcDNA3.1, pcDNA-RP11-499E18.1, and pcDNA-RP11-499E18.1 + pcDNA-PAK2 were respectively, transfected into CaOV3 cells. Then, the transfected cells were respectively inoculated at the right dorsal proximal upper limbs of nude mice. **(A,B)** RP11-499E18.1 overexpression remarkably inhibited tumor growth as time prolongs, while PAK2 upregulation notably reversed this effect. **(C,D)** RP11-499E18.1 overexpression remarkably increased the expression of apoptosis-related Caspase 3, while notably decreased the expression of proliferation-associated Ki67. These effects were notably counteracted by PAK2 upregulation. PAK2, P21 (RAC1) activated kinase 2. ^#^*p* < 0.05.

To verify the effect of RP11-499E18.1 and PAK2 overexpression on tumor development, we implanted pcDNA-RP11-499E18.1 or pcDNA-RP11-499E18.1 plus pcDNA-PAK2 transfected CaOV3 cells into the nude mice. Tumor volume measurement results demonstrated that RP11-499E18.1 overexpression distinctly inhibited tumor development of CaOV3 cells as time prolongs, while PAK2 upregulation notably reversed this effect (Figure 8A, both *p* < 0.001). These effects could be obviously observed from the final tumor volume formatted in nude mice (Figure 8B). In addition, expression of proliferation-related Ki67 and apoptosis-associated Caspase 3 was determined employing IHC assay. Outcomes showed that the expression of Ki67 was notably reduced, while Caspase 3 expression was remarkably augmented by RP11-499E18.1 overexpression compared with the pcDNA transfected group. Whereas PAK2 upregulation remarkably decreased Caspase 3 expression, it notably increased Ki67 expression compared to the pcDNA-RP11-499E18.1 plus pcDNA-PAK2 transfected group (Figures 8C,D, all *p* < 0.05). These results further validated the tumor volume outcomes, indicating that RP11-499E18.1 overexpression might carry out its tumor-suppressing effects by increasing the expression of apoptosis-related gene expression and decreasing the expression of proliferation-associated gene expression.

## Discussion

Ovarian cancer is a deadly gynecological malignancy in the world. In recent years, a number of former investigations have discovered the participation of lncRNAs in the regulation of OC progression with the help of bioinformatic analysis (Wu et al., 2017; Li et al., 2018). In this research, we identified lncRNA RP11-499E18.1, which was downregulated in OC tissues, as a potential biomarker of OC *via* GEO database screening and prognostic analysis. Then, GEO database analysis combined with GO term analysis discovered that PAK2 might be the critical mRNA that participated in the regulation of RP11-499E18.1, while literature analysis found that SOX2 could be the target protein of PAK2. After that, experimental analysis verified that there was an interaction between RP11-499E18.1 and PAK2, as well as PAK2 and SOX2. Therefore, we speculated that RP11-499E18.1 might play its tumor suppressor roles in OC *via* regulation of the RP11-499E18.1–PAK2–SOX2 axis.

Obviously, we discovered that RP11-499E18.1 functions as a tumor suppressor in OC exhibiting as RP11-499E18.1 overexpression, which notably suppressed the proliferation, migration, and EMT process of OC cells. These outcomes were consistent with former researches. For instance, it was demonstrated that overexpression of Titin-antisense RNA1 (TTN-AS1) repressed colony formation and proliferation, while facilitating apoptosis of OC cells (Miao et al., 2020). Besides, Ma et al. (2018) reported that decreased expression of growth arrest-specific transcript 5 (GAS5) was correlated with advanced clinical stage, and overexpression of GAS5 obviously suppressed the proliferation of OC cells. Moreover, Tao et al. (2020) clarified that maternally expressed 3 gene (MEG3) overexpression notably promoted apoptosis, while restraining the viability and invasion of OC cells.

LncRNAs have been widely proved to be the controller of their downstream targets in OC (Liang et al., 2018; Chen X. et al., 2019; Sun et al., 2019; Yang et al., 2019). For example, Kong et al. (2019) clarified that lncRNA PCAT6 facilitated the initiation and development of OC through repressing the expression of PTEN. Another research demonstrated that lncRNA LINC00702 accelerated the development of OC by interacting with EZH2 and thereby repressing the transcription of KLF2 (Wang L. et al., 2019). Herein, this research identified PAK2 as a target of RP11-499E18.1 and proved that there was an interaction between RP11-499E18.1 and PAK2. Besides, PAK2 upregulation partially reversed RP11-499E18.1 overexpression-triggered tumor-suppressing effects on OC cells. However, RP11-499E18.1 overexpression did not alter the expression of PAK2, so we deduced that RP11-499E18.1 might function in OC through affecting the interaction between PAK2 and its target and thereby influencing the downstream gene transcription.

Considering that PAK2 is a member of the serine/threonine kinases family, this study subsequently focused on the target effect of PAK2 kinase and further searched for the potential PAK2 kinase target proteins. Former investigations pointed out that SOX2 was an interacting protein of PAK2 (Huttlin et al., 2015, 2017). Besides, SOX2 was reported to be associated with early tumor initiation (Robinson et al., 2021), and its level in high-grade serous carcinoma (HGSC) effusions was demonstrated to be markers of clinically aggressive disease (Sherman-Samis et al., 2019). Additionally, SOX2 expression was found to be positively correlated with the proliferation and migration capacities of tumor cells (Chen B. et al., 2019; Meng et al., 2020), and its high expression is a poor prognostic marker for OC. Furthermore, it was clarified that the interaction between SOX2 and UBR5 was inhibited after phosphorylation of SOX2, and thereby stabilized SOX2 in esophageal cancer (Wang Z. et al., 2019). Moreover, CDK1 knockdown decreased the phosphorylation, transcription activity, and nuclear distribution of SOX2, while knockout of SOX2 significantly reduced CDK1 overexpression-induced tumor-initiating capacity (Ravindran Menon et al., 2018). Based on these evidences, we speculated that PAK2 upregulation might perform its tumor-promoting efficacy through increasing the phosphorylation and nuclear distribution of SOX2 in OC cells. Following results confirmed this speculation. Additionally, knockdown of SOX2 notably counteracted PAK2 upregulation-induced tumor-promoting effects on cell proliferation, migration, and expression of EMT-related factors.

Previous investigation demonstrated that the core of personalized medicine for cancer lies in the discovery and development of biomarkers, which reveal information that leads to the diagnosis, prognosis, and therapy of the disease (Dlamini et al., 2021). Besides, a previous research pointed out that uncertainty of prognosis is the main reason that patients with hematologic malignancies receive aggressive therapy near end of life (Iizuka-Honma et al., 2021). In OC, patients are generally diagnosed at an advanced stage primarily because there are few early symptoms and limited screening approaches. Therefore, we hold the opinion that identification of novel effective diagnostic biomarkers, like RP11-499E18.1, will be of great significance for better management of OC in the future.

## Conclusion

**FIGURE 9:**
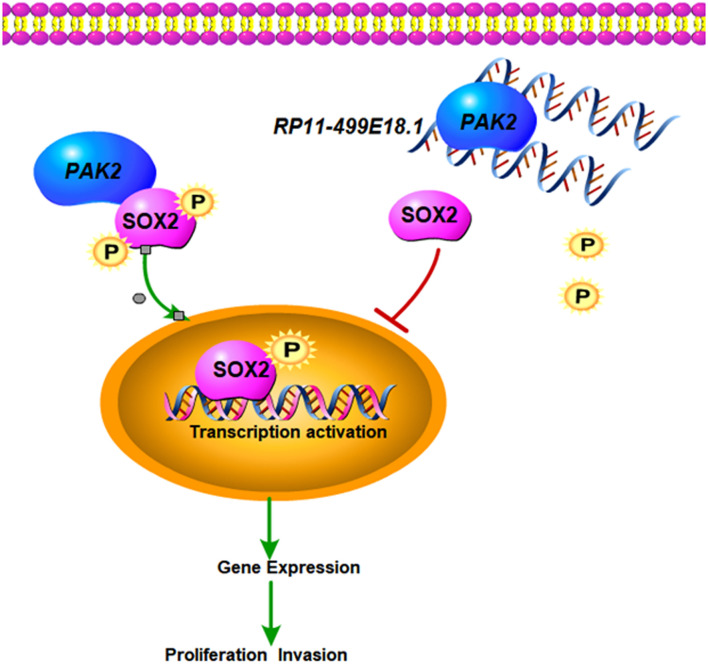
The putative regulation network of this study. Upregulated RP11-499E18.1 inhibits the proliferation, migration, colony formation, and EMT process of OC cells through dissociating the interaction between PAK2 and SOX2 and reducing the nuclear translocation of p-SOX2.

Our results demonstrated that RP11-499E18.1 overexpression might have inhibited proliferation, migration, colony formation, and EMT process of OC cells through dissociating the interaction between PAK2 and SOX2 and reducing the nuclear translocation of p-SOX2. This research mainly explored the potential regulating mechanisms of RP11-499E18.1 on OC progression *in vitro*, but whether it functions through the same pattern *in vivo* still needs further investigation. The putative regulation network is displayed in Figure 9.

## Data Availability Statement

The original contributions presented in the study are included in the article/Supplementary Material, further inquiries can be directed to the corresponding authors.

## Ethics Statement

The studies involving human participants were reviewed and approved by Ethics Committee of Medical ethics committee of Hunan Cancer Hospital. The patients/participants provided their written informed consent to participate in this study. The animal study was reviewed and approved by Medical ethics committee of Hunan Cancer Hospital. Written informed consent was obtained from the owners for the participation of their animals in this study.

## Author Contributions

JY and SP conceived and designed the study. JY, KZ, and SP performed the experiments and interpreted the data. JY collected the data. KZ and SP analyzed the data and wrote the manuscript. All authors reviewed the results and approved the final version of the manuscript.

## Conflict of Interest

The authors declare that the research was conducted in the absence of any commercial or financial relationships that could be construed as a potential conflict of interest.

## Publisher’s Note

All claims expressed in this article are solely those of the authors and do not necessarily represent those of their affiliated organizations, or those of the publisher, the editors and the reviewers. Any product that may be evaluated in this article, or claim that may be made by its manufacturer, is not guaranteed or endorsed by the publisher.

## Footnotes

1
https://www.ncbi.nlm.nih.gov/geo/

2
https://www.ncbi.nlm.nih.gov/geo/geo2r/

3
https://david.ncifcrf.gov/

4
http://service.tartaglialab.com/page/catrapid_group

## References

[B1] Allemani C. Matsuda T. Di Carlo V. Harewood R. Matz M. Nikšić M. (2018). Global surveillance of trends in cancer survival 2000–14 (CONCORD-3): analysis of individual records for 37 513 025 patients diagnosed with one of 18 cancers from 322 population-based registries in 71 countries. *Lancet* 391 1023–1075. 29395269 10.1016/S0140-6736(17)33326-3 PMC5879496

[B2] Arnes L. Liu Z. Wang J. Maurer C. Sagalovskiy I. Sanchez-Martin M. (2019). Comprehensive characterisation of compartment-specific long non-coding RNAs associated with pancreatic ductal adenocarcinoma. *Gut* 68 499–511. 10.1136/gutjnl-2017-314353 29440233 PMC6086768

[B3] Au K. K. Josahkian J. A. Francis J. A. Squire J. A. Koti M. (2015). Current state of biomarkers in ovarian cancer prognosis. *Future Oncol.* 11 3187–3195. 10.2217/fon.15.251 26551891

[B4] Bill M. Papaioannou D. Karunasiri M. Kohlschmidt J. Pepe F. Walker C. J. (2019). Expression and functional relevance of long non-coding RNAs in acute myeloid leukemia stem cells. *Leukemia* 33 2169–2182. 10.1038/s41375-019-0429-5 30858548

[B5] Chen B. Zhu Z. Li L. Ye W. Zeng J. Gao J. (2019). Effect of overexpression of Oct4 and Sox2 genes on the biological and oncological characteristics of gastric cancer cells. *Onco Targets Ther.* 12 4667–4682. 10.2147/ott.s209734 31417271 PMC6592062

[B6] Chen J. Cai Y. Zhou J. Mei J. (2020). Identification of four hub genes as promising biomarkers to evaluate the prognosis of ovarian cancer in silico. *Cancer Cell Int.* 20 1–11. 32595417 10.1186/s12935-020-01361-1 PMC7315561

[B7] Chen X. Wu W. Cao X. Zhao X. Li W. Deng C. (2019). lncRNA mortal obligate RNA transcript was downregulated in ovarian carcinoma and inhibits cancer cell proliferation by downregulating miRNA-21. *J. Cell. Biochem.* 120 11949–11954. 10.1002/jcb.28478 30916806

[B8] Cress R. D. Chen Y. S. Morris C. R. Petersen M. Leiserowitz G. S. (2015). Characteristics of long-term survivors of epithelial ovarian cancer. *Obstetr. Gynecol.* 126:491. 10.1097/aog.0000000000000981 26244529 PMC4545401

[B9] Dlamini Z. Mbele M. Makhafola T. J. Hull R. Marima R. (2021). HIV-Associated Cancer Biomarkers: A Requirement for Early Diagnosis. *Int. J. Mol. Sci.* 22:8127. 10.3390/ijms22158127 34360891 PMC8348540

[B10] Gutschner T. Diederichs S. (2012). The hallmarks of cancer: a long non-coding RNA point of view. *RNA Biol.* 9 703–719. 10.4161/rna.20481 22664915 PMC3495743

[B11] Hao T. Huang S. Han F. (2020). LINC-PINT suppresses tumour cell proliferation, migration and invasion through targeting miR-374a-5p in ovarian cancer. *Cell Biochem. Funct.* 38 1089–1099. 10.1002/cbf.3565 32638404

[B12] Hosono Y. Niknafs Y. S. Prensner J. R. Iyer M. K. Dhanasekaran S. M. Mehra R. (2017). Oncogenic Role of THOR, a Conserved Cancer/Testis Long Non-coding RNA. *Cell* 171 1559.e–1572.e. 29245011 10.1016/j.cell.2017.11.040 PMC5734106

[B13] Huarte M. (2015). The emerging role of lncRNAs in cancer. *Nat. Med.* 21 1253–1261. 10.1038/nm.3981 26540387

[B14] Huttlin E. L. Bruckner R. J. Paulo J. A. Cannon J. R. Ting L. Baltier K. (2017). Architecture of the human interactome defines protein communities and disease networks. *Nature* 545 505–509. 10.1038/nature22366 28514442 PMC5531611

[B15] Huttlin E. L. Ting L. Bruckner R. J. Gebreab F. Gygi M. P. Szpyt J. (2015). The BioPlex network: a systematic exploration of the human interactome. *Cell* 162 425–440. 10.1016/j.cell.2015.06.043 26186194 PMC4617211

[B16] Iizuka-Honma H. Mitsumori T. Yoshikawa S. Takizawa H. Noguchi M. (2021). Prognostic Value of Palliative Prognostic Index for Hospitalized Patients With End-of-Life Hematologic Malignancies in a Japanese University Hospital. *JCO Oncol. Pract.* 2021:O2100243. 10.1200/OP.21.00243 34357786

[B17] Kong F. R. Lv Y. H. Yao H. M. Zhang H. Y. Zhou Y. Liu S. E. (2019). LncRNA PCAT6 promotes occurrence and development of ovarian cancer by inhibiting PTEN. *Eur. Rev. Med. Pharmacol. Sci.* 23 8230–8238. 31646553 10.26355/eurrev_201910_19132

[B18] Kossaï M. Leary A. Scoazec J. Y. Genestie C. (2018). Ovarian cancer: a heterogeneous disease. *Pathobiology* 85 41–49. 29020678 10.1159/000479006

[B19] Li N. Zhan X. Zhan X. (2018). The lncRNA SNHG3 regulates energy metabolism of ovarian cancer by an analysis of mitochondrial proteomes. *Gynecol. Oncol.* 150 343–354. 10.1016/j.ygyno.2018.06.013 29921511

[B20] Liang H. Yu T. Han Y. Jiang H. Wang C. You T. (2018). LncRNA PTAR promotes EMT and invasion-metastasis in serous ovarian cancer by competitively binding miR-101-3p to regulate ZEB1 expression. *Mol. Cancer* 17 1–13. 30098599 10.1186/s12943-018-0870-5 PMC6087007

[B21] Livak K. J. Schmittgen T. D. (2001). Analysis of relative gene expression data using real-time quantitative PCR and the 2- ΔΔCT method. *Methods* 25 402–408. 10.1006/meth.2001.1262 11846609

[B22] Ma N. Li S. Zhang Q. Wang H. Qin H. Wang S. (2018). Long non-coding RNA GAS5 inhibits ovarian cancer cell proliferation via the control of microRNA-21 and SPRY2 expression. *Exp. Therapeut. Med.* 16 73–82. 10.3892/etm.2018.6188 PMC5995084 29896229

[B23] Meng Y. Xu Q. Chen L. Wang L. Hu X. (2020). The function of SOX2 in breast cancer and relevant signaling pathway. *Pathol. Res. Pract.* 216:153023. 10.1016/j.prp.2020.153023 32703490

[B24] Menon U. Karpinskyj C. Gentry-Maharaj A. (2018). Ovarian cancer prevention and screening. *Obstetr. Gynecol.* 131 909–927. 10.1097/AOG.0000000000002580 29630008

[B25] Miao S. Wang J. Xuan L. Liu X. LncRNA T. T. N. (2020). AS1 acts as sponge for miR-15b-5p to regulate FBXW7 expression in ovarian cancer. *BioFactors* 46 600–607. 10.1002/biof.1622 32049388

[B26] Nagano T. Fraser P. (2011). No-nonsense functions for long noncoding RNAs. *Cell* 145 178–181. 10.1016/j.cell.2011.03.014 21496640

[B27] Ravindran Menon D. Luo Y. Arcaroli J. J. Liu S. KrishnanKutty L. N. Osborne D. G. (2018). CDK1 interacts with Sox2 and promotes tumor initiation in human melanoma. *Cancer Res.* 78 6561–6574. 10.1158/0008-5472.can-18-0330 30297536 PMC6279496

[B28] Robinson M. Gilbert S. F. Waters J. A. Lujano-Olazaba O. Lara J. Alexander L. J. (2021). Characterization of SOX2, OCT4 and NANOG in Ovarian Cancer Tumor-Initiating Cells. *Cancers* 13:262. 10.3390/cancers13020262 33445692 PMC7828139

[B29] Schmitt A. M. Chang H. Y. (2016). Long Noncoding RNAs in Cancer Pathways. *Cancer Cell* 29 452–463. 10.1016/j.ccell.2016.03.010 27070700 PMC4831138

[B30] Sherman-Samis M. Onallah H. Holth A. Reich R. Davidson B. (2019). SOX2 and SOX9 are markers of clinically aggressive disease in metastatic high-grade serous carcinoma. *Gynecol. Oncol.* 153 651–660. 10.1016/j.ygyno.2019.03.099 30904337

[B31] Sun Q. Li Q. Xie F. (2019). LncRNA-MALAT1 regulates proliferation and apoptosis of ovarian cancer cells by targeting miR-503-5p. *OncoTargets Therapy* 12:6297. 10.2147/ott.s214689 31496733 PMC6691960

[B32] Sundar S. Neal R. D. Kehoe S. (2015). Diagnosis of ovarian cancer. *BMJ* 2015:351. 10.1136/bmj.h4443 26328593

[B33] Tang X. J. Wang W. Hann S. S. (2019). Interactions among lncRNAs, miRNAs and mRNA in colorectal cancer. *Biochimie* 163 58–72. 10.1016/j.biochi.2019.05.010 31082429

[B34] Tao P. Yang B. Zhang H. Sun L. Wang Y. Zheng W. (2020). The overexpression of lncRNA MEG3 inhibits cell viability and invasion and promotes apoptosis in ovarian cancer by sponging miR-205-5p. *Int. J. Clin. Exp. Pathol.* 13:869. PMC7270692 32509057

[B35] Wang L. Ye T. Y. Wu H. Chen S. Y. Weng J. R. Xi X. W. (2019). LINC00702 accelerates the progression of ovarian cancer through interacting with EZH2 to inhibit the transcription of KLF2. *Eur. Rev. Med. Pharmacol. Sci.* 23(3 Suppl.), 201–208. 10.26355/eurrev_201908_18648 31389610

[B36] Wang Y. Zhang M. Zhou F. (2020). Biological functions and clinical applications of exosomal long non-coding RNAs in cancer. *J. Cell Mol. Med.* 24 11656–11666. 10.1111/jcmm.15873 32924276 PMC7578871

[B37] Wang Z. Kang L. Zhang H. Huang Y. Fang L. Li M. (2019). AKT drives SOX2 overexpression and cancer cell stemness in esophageal cancer by protecting SOX2 from UBR5-mediated degradation. *Oncogene* 38 5250–5264. 10.1038/s41388-019-0790-x 30894683

[B38] Wu D. D. Chen X. Sun K. X. Wang L. L. Chen S. Zhao Y. (2017). Role of the lncRNA ABHD11-AS(1) in the tumorigenesis and progression of epithelial ovarian cancer through targeted regulation of RhoC. *Mol. Cancer* 16:138. 10.1186/s12943-017-0709-5 PMC5561620 28818073

[B39] Xu C. Zhu L. X. Sun D. M. Yao H. Han D. X. (2020). Regulatory mechanism of lncRNA NORAD on proliferation and invasion of ovarian cancer cells through miR-199a-3p. *Eur. Rev. Med. Pharmacol. Sci.* 24 1672–1681. 32141533 10.26355/eurrev_202002_20341

[B40] Yang M. Zhai Z. Guo S. Li X. Zhu Y. Wang Y. (2019). Long non-coding RNA FLJ33360 participates in ovarian cancer progression by sponging miR-30b-3p. *OncoTargets Therapy* 12:4469. 10.2147/ott.s205622 31239715 PMC6560195

[B41] Zhang Y. B. Jiang Y. Wang J. Ma J. Han S. (2019). Evaluation of core serous epithelial ovarian cancer genes as potential prognostic markers and indicators of the underlying molecular mechanisms using an integrated bioinformatics analysis. *Oncol. Lett.* 18 5508–5522. 31612059 10.3892/ol.2019.10884 PMC6781641

