# Supplementary material for: LncRNA RP11-499E18.1 Inhibits Proliferation, Migration, and Epithelial–Mesenchymal Transition Process of Ovarian Cancer Cells by Dissociating PAK2–SOX2 Interaction
Source: Front Cell Dev Biol. 2021 Sep 21;9:697831. doi: 10.3389/fcell.2021.697831 (PMC8490721; doi:10.3389/fcell.2021.697831)
Supplement: Supplementary file 2 [file Data_Sheet_2.DOCX]

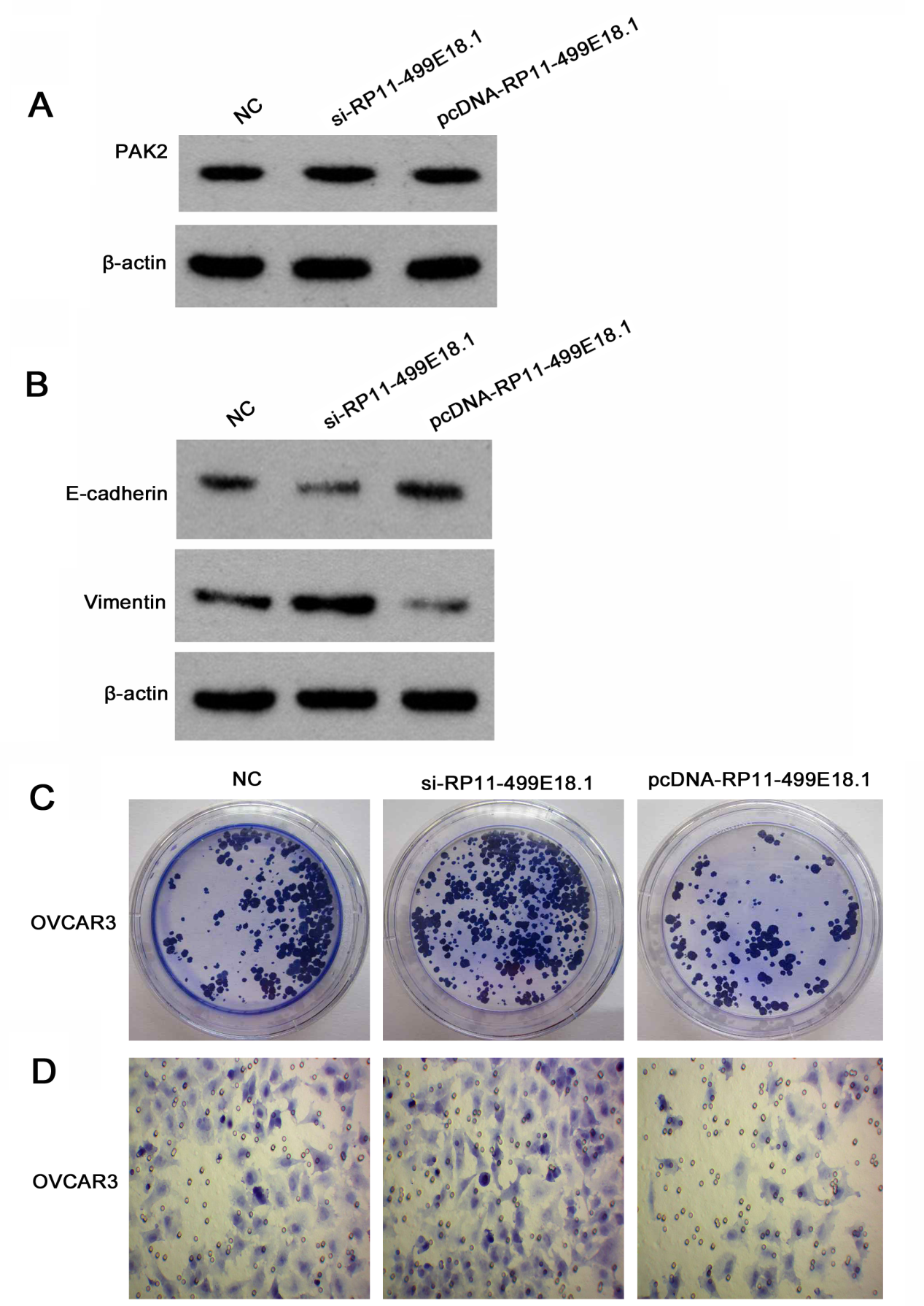


**Supplementary figure 1. RP11-499E18.1 overexpression obviously suppressed cell migration, colony formation and EMT transformation**

The sequences of NC and si-RP11-499E18.1 were respectively transfected into OVCAR3 cells to silence RP11-499E18.1 expression. The plasmids pcDNA 3.1 and pcDNA-RP11-499E18.1 were respectively transfected into OVCAR3 cells to overexpress RP11-499E18.1. A, western blot assay was conducted to detect PAK2 protein level. B, western blot assay was conducted to detect EMT markers, E-cadherin and Vimentin. C and D, cell migration and invasion. NC, negative control; si, small interfering; PAK2, P21 (RAC1) Activated Kinase 2; EMT, epithelial-mesenchymal transition.


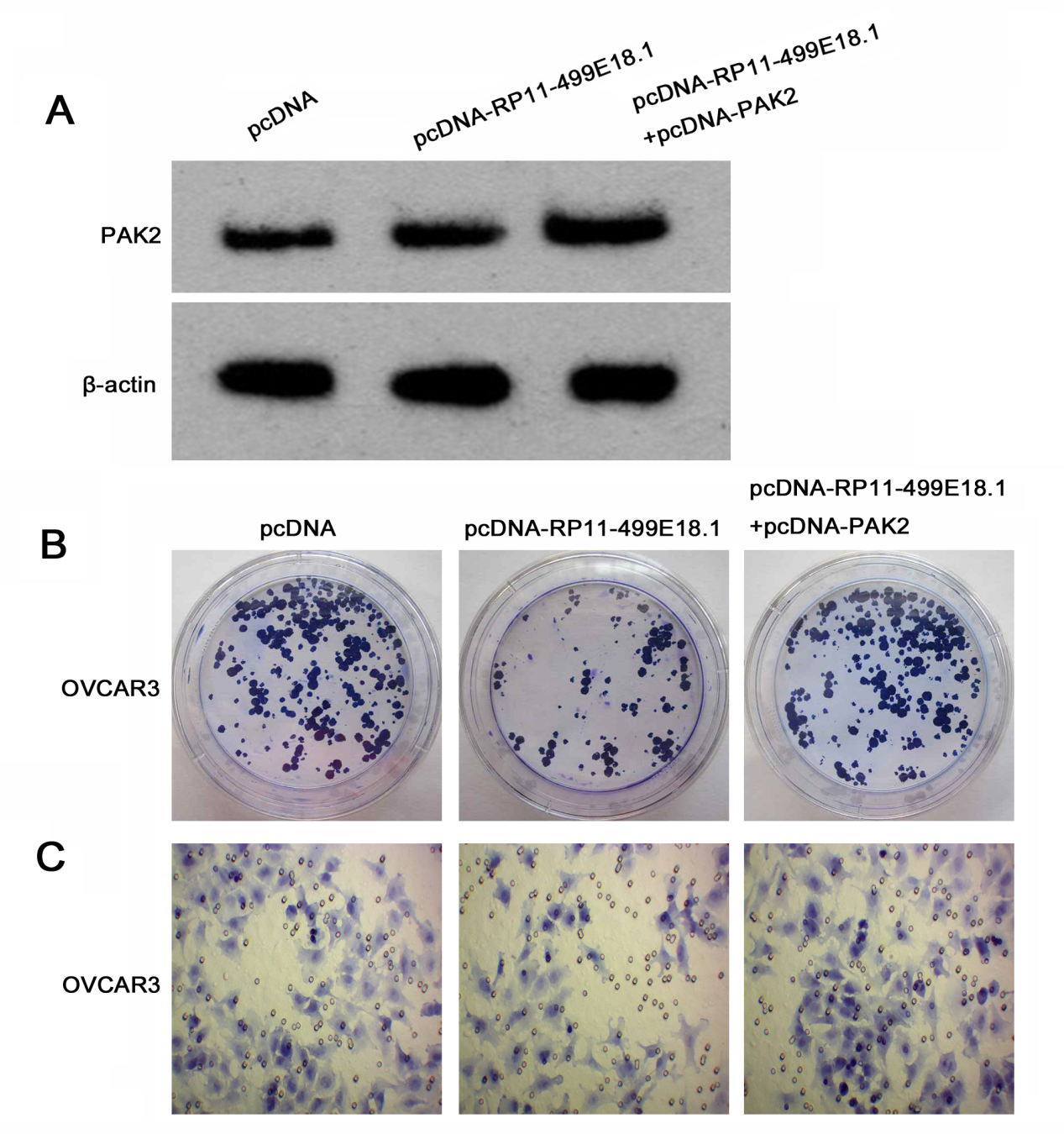


**Supplementary Figure 2. PAK2 upregulation notably counteracted RP11-499E18.1 overexpression-triggered tumor suppressing effects**

The plasmids pcDNA3.1, pcDNA-RP11-499E18.1 and pcDNA-RP11-499E18.1 + pcDNA-PAK2 were respectively transfected into OVCAR3 cells. A, western blot assay was conducted to detect PAK2 protein level. B and C, cell migration and invasion.PAK2, P21 (RAC1) Activated Kinase 2.


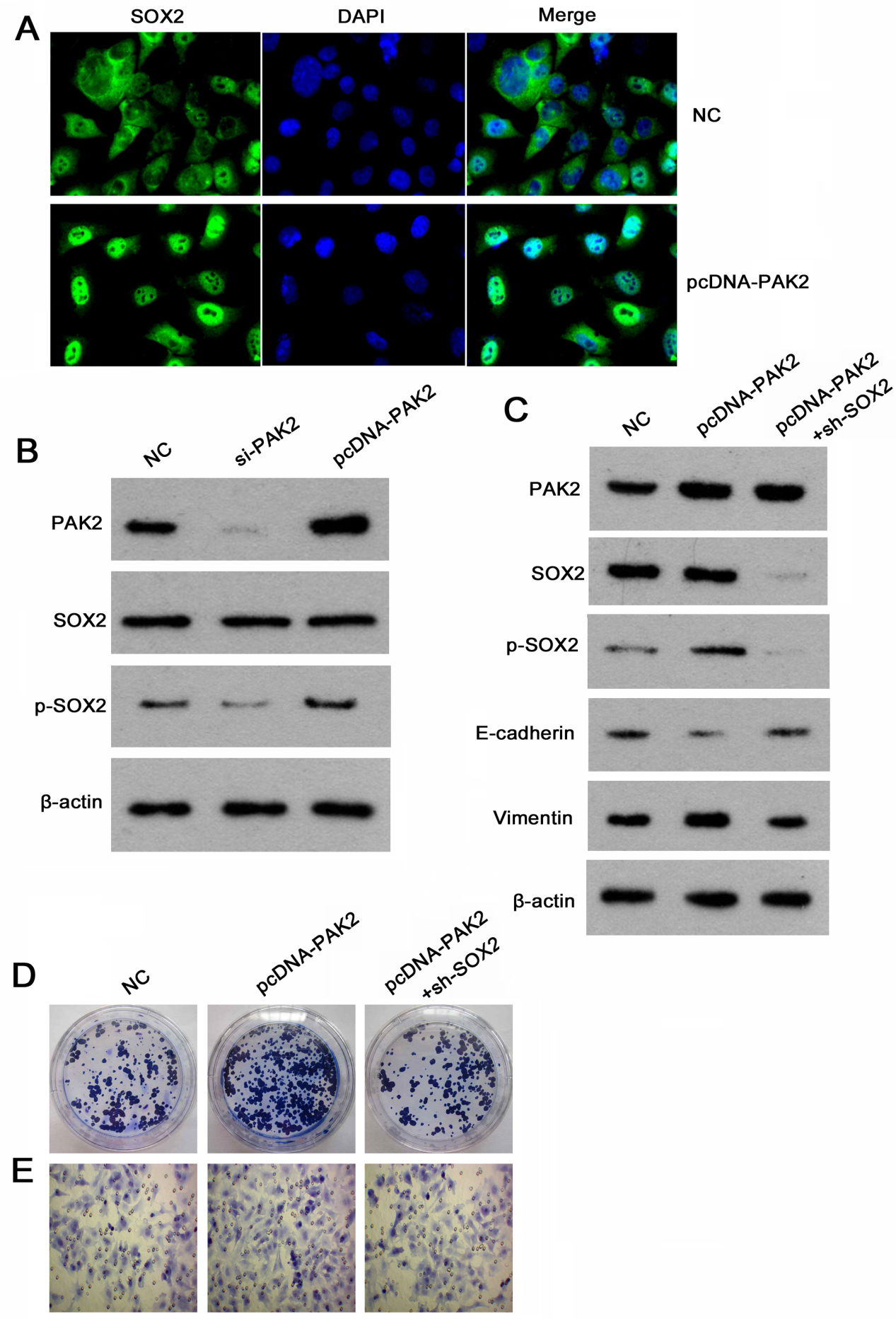


**Supplementary Figure 3. Knockdown of SOX2 notably reversed PAK2 overexpression-triggered tumor promoting effects.**

The plasmids NC, si-PAK2, pcDNA-PAK2 and pcDNA-PAK2+sh-SOX2 were transfected into OVCAR3 cells. A, IF assay was conducted to detect SOX2 in OVCAR3 cells. B, western blot assay was conducted to detect PAK2, SOX2 and p-SOX2 protein levels. C, western blot assay was conducted to detect PAK2, SOX2, p-SOX2, E-cadherin and Vimentin. D and E, cell migration and invasion. PAK2, P21 (RAC1) Activated Kinase 2; SOX2, SRY-Box Transcription Factor 2; EMT, epithelial-mesenchymal transition.
